# Recent Progress in Gene Therapy for Ovarian Cancer

**DOI:** 10.3390/ijms19071930

**Published:** 2018-06-30

**Authors:** Ángela Áyen, Yaiza Jiménez Martínez, Juan A. Marchal, Houria Boulaiz

**Affiliations:** 1 Department of Human Anatomy and Embryology, University of Granada, 18016 Granada, Spain; aayen@correo.ugr.es (A.A.); jmarchal@ugr.es (J.A.M.); 2 Biopathology and Medicine Regenerative Institute (IBIMER), University of Granada, 18016 Granada, Spain; yaijmartinez@correo.ugr.es; 3 Biosanitary Institute of Granada (ibs.GRANADA), SAS-Universidad de Granada, 18016 Granada, Spain; 4 Excellence Research Unit “Modeling Nature” (MNat), University of Granada, 18016 Granada, Spain

**Keywords:** ovarian cancer, gene therapy, delivery systems, promoter, suicide genes, ovarian cancer stem cells

## Abstract

Ovarian cancer is the most lethal gynecological malignancy in developed countries. This is due to the lack of specific symptoms that hinder early diagnosis and to the high relapse rate after treatment with radical surgery and chemotherapy. Hence, novel therapeutic modalities to improve clinical outcomes in ovarian malignancy are needed. Progress in gene therapy has allowed the development of several strategies against ovarian cancer. Most are focused on the design of improved vectors to enhance gene delivery on the one hand, and, on the other hand, on the development of new therapeutic tools based on the restoration or destruction of a deregulated gene, the use of suicide genes, genetic immunopotentiation, the inhibition of tumour angiogenesis, the alteration of pharmacological resistance, and oncolytic virotherapy. In the present manuscript, we review the recent advances made in gene therapy for ovarian cancer, highlighting the latest clinical trials experience, the current challenges and future perspectives.

## 1. Introduction

Ovarian cancer (OC) is the seventh most frequent cancer among women worldwide with an incidence of 238.700 new cases and 151.900 annual deaths [1]. In developed countries, it is the second most common gynaecological cancer (99.800 new cases) and the most lethal (65.000 deaths), since the majority of OC are diagnosed at an advanced stage [1]. OC must be considered as a group of different diseases with differences in epidemiologic and risk factors, premalignant lesions (serous tubal intraepithelial neoplasia or endometriosis), molecular events, response to chemotherapy and prognosis. Primary fallopian tube cancer and peritoneal serous carcinoma are considered rare malignancies, however, many tumours that were classified as serous carcinomas of the ovary or peritoneal cancers appear to have their origin in the fallopian tube [2]. These carcinomas have similarities in histology, genetic and clinical behaviour with OC, so they should be considered collectively and are managed similarly [3]. The International Federation of Gynecology and Obstetrics (FIGO) staging system combined ovarian, fallopian tube, and peritoneal cancers into a single classification in 2014, based on surgical stage [4].

Based on clinical, histologic and molecular factors, epithelial ovarian cancer (EOC) can be divided into two subtypes: Type I and Type II. Type I tumors are slow growing, remain confined to the ovary for long periods and have an indolent course. They include (i) endometriosis-related tumors (endometrioid, clear cell, and seromucinous carcinomas); (ii) low-grade serous carcinomas; and (iii) mucinous carcinomas and malignant Brenner tumors. In contrast, type II tumors are high-grade, spread rapidly and are highly aggressive at their onset. They include high-grade serous carcinoma (poorly and moderately differentiated), carcinosarcomas (malignant mixed mesodermal tumors) and undifferentiated carcinoma [5].

### 1.1. Risk Factors and Prevention Strategies

The risk of OC is increased in women with a family history of OC, personal history of breast cancer, mutation in BRCA1 or BRCA2, Lynch syndrome, increased age, infertility, nulliparity, hormonal factors like early age at menarche or late age at menopause, inflammatory states such as endometriosis or pelvic inflammatory disease, and obesity. In contrast, multiparity, history of breastfeeding, taking oral contraceptives, hysterectomy and tubal ligation appear to have a protective role [6]. The risk factors for the fallopian tube and peritoneal carcinoma are unclear [7].

### 1.2. Diagnostic and Early Detection

The clinical presentation of EOC is commonly insidious, making diagnosis at an early stage more difficult. The majority of women have stage III or stage IV at diagnosis, and present abdominal pain or discomfort, menstrual irregularities, dyspepsia and other gastrointestinal symptoms, and urinary symptoms of frequency or retention. The advanced disease respiratory symptoms appear from ascites or pleural effusion, and bowel obstruction [3].

Most cases of OC are diagnosed at later stages, with a high mortality. There is no adequate screening test for ovarian cancer in asymptomatic women without high risk of developing this pathology, since strategies based on measurement of serum CA-125 concentration, transvaginal ultrasound, or both, are not sensitive enough to early stage detection of this disease. Moreover, they generate a negative balance between the important harm derived from false positives and the number of OCs detected [8]. In a recent study, a panel of four markers, CA-125, HE4, E-CAD and IL-6, was selected and showed great potential in the detection of high-grade serous ovarian carcinoma at earlier stages in samples collected from 172 patients. However, additional validation studies using the combination of biomarkers in patients with OC are needed to confirm its effectiveness [9]. In addition, woman with a suspected high-risk hereditary cancer syndrome (BRCA1 and BRCA2 mutation and Lynch syndrome) should receive genetic counselling, and, if the mutation is confirmed, to consider prophylactic surgery (risk-reducing bilateral salpingo-oophorectomy [3]).

**Figure 1:**
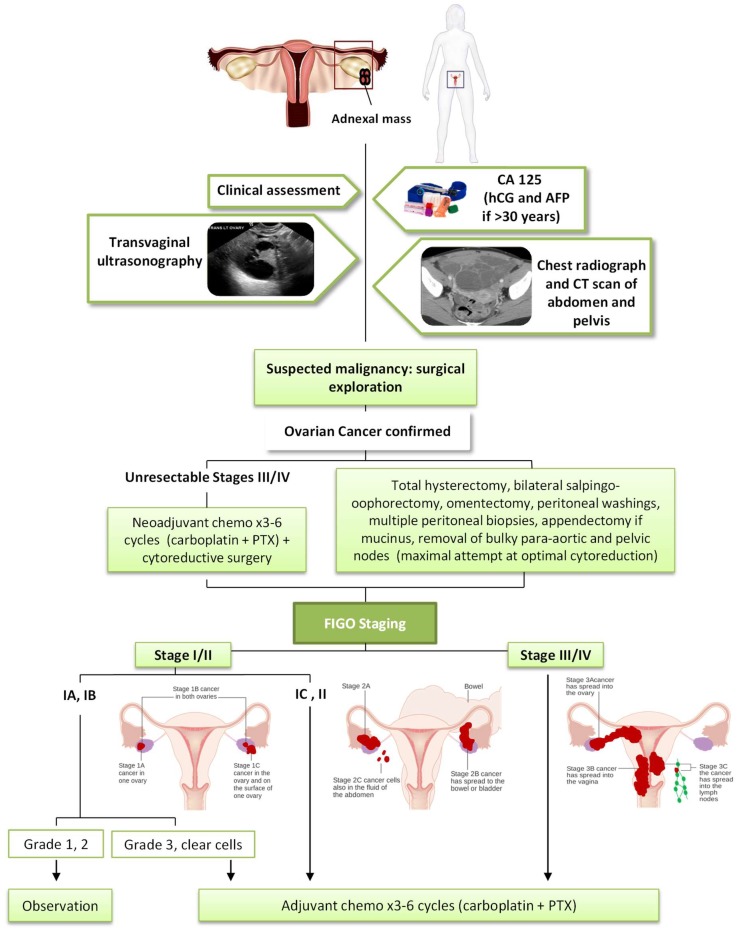
Algorithm for management of ovarian cancer. Figo Staging ovarian images modified from Cancer Research UK/Wikimedia Commons.

As shown in Figure 1, the histologic diagnosis, stage and prognosis of epithelial ovarian, fallopian tube or peritoneal cancer require surgical exploration. A clinical assessment and measurement of serum CA-125 aids diagnosis. Human gonadotropin (hCG) and alpha-fetoprotein (AFP) allow us to exclude the origin in the germ cell. Transvaginal ultrasonography is the ideal imaging investigation in the visualisation of ovarian masses, allowing us to see characteristics suggestive of malignancy (International Ovarian Tumor Analysis (IOTA) simple rules) [10]. Prior to surgery, radiographers should take a chest X-Ray and a CT scan of the abdomen and pelvis to evaluate the presence of metastatic disease. Cancer of the ovary, fallopian tube, or peritoneum is staged according to the 2017 FIGO staging system [3].

### 1.3. Current Treatments of Ovarian Cancer

The current standard of care for EOC is an optimal cytoreductive surgery followed by a combination of chemotherapy with a platinum and taxane regimen (Figure 1). The most important prognostic indicator in patients with advanced stage is the volume of residual disease after surgery, so these patients should undergo a total hysterectomy, bilateral salpingo-oophorectomy, omentectomy, and a maximal attempt at optimal cytoreduction. In addition, peritoneal washings, multiple peritoneal biopsies, appendectomy in mucinous histology and removal of bulky para-aortic and pelvic nodes are performed. In some patients with advanced stage (IIIC or IV) and unresectable tumours it is necessary for 2–3 cycles of neoadjuvant chemotherapy initially, followed by surgical cytoreduction and additional chemotherapy. This approach may also be used in a patient with a primary suboptimal cytoreduction [3,10].

In relation to adjuvant chemotherapy in an early stage, it is not indicated in stage IA and IB grade 1–2, but it offers benefits in patients with grade 3 of differentiation, stage IC and II or clear-cell histology, in which 3–6 cycles of carboplatin and paclitaxel (PTX) are administered. Chemotherapy is recommended for all patients with stage II-IV, concretely 6 cycles of carboplatin and PTX (or docetaxel if PTX is not tolerated) [3,10].

Despite an adequate approach, the majority of women with advanced-stage OC will relapse due to platinum-resistant or refractory cancer, with a median time to recurrence of 16 months [3]. Thus, there has been significant interest in developing innovative strategies more targeted at treating this pathology and gene therapy represents a good therapeutic option. In this review, we will summarize the latest advances in the application of gene therapy in OC, providing a basic understanding of current vector technology, possible relevant genetic targets and a summary of clinical trials.

## 2. Gene Therapy in Ovarian Cancer

Although gene therapy for OC is in continuous progress and innovation, it is far from reaching the patient. Hence, several strategies have been developed to improve the performance of these systems.

### 2.1. Improved Vectors for Gene Delivery

One of the limitations of clinical success in gene therapy is still the lack of a safe and highly efficient gene delivery system. An optimal vector for gene therapy should selectively target the tumour cells allowing (i) an improvement in transfection efficiencies; (ii) minimization of off-target transfection; and (iii) reduction of genotoxicity, which have long been recognized as the major obstacles of gene therapy [11]. In this context, many efforts are being made and a wide range of viral and non-viral vectors have been developed.

#### 2.1.1. Viral Vectors

##### Multiple Viral Vectors Have Been Evaluated in OC

Lentiviruses are one of the most studied vectors for targeted gene therapy due to their ability to transduce both dividing and non-dividing cells and allow long-term transgene expression in vivo and in vitro through integration into host cell genomes. Huhtala et al. used cetuximab (anti-epidermal growth factor receptor (EGFR) antibody)-conjugated lentivirus vectors to improve the effectiveness of the treatment in nude mice with orthotropic SKOV-3m human ovarian carcinoma xenografts. This vector induced a significant antitumour immunity leading to tumour regression [12].

Adenoviral vectors (ADV) infect both dividing and non-dividing cells, are safe, have a large cloning capacity and facilitate gene expression in 12 h after infection. However, their capacity to infect cells is dependent upon the presence of Coxsackievirus and adenovirus receptors (CAR) that have a low concentration in target tumours including OC, meaning a restricted transfection efficacy. To avoid this limitation, Rawlinson et al. incorporated an Arg-Gly-Asp peptide (RGD) that allows the virus to use an alternative receptor during transduction and increased its efficiency [13]. Another problem of the recombinant adenovirus is that repeated administration will develop an immune response, with neutralization of the adenovirus by antibodies. The use of protective polymer-coating on the virus particles can avoid this problem. Yoshihara et al. coated adenoviruses by layer-on-layer deposition of ionic polymers (polyethyleneimine (PEI) and hyaluronic acid) onto adenovirus particles to produce multilayer-coated virus vectors. They reported that the infectivity of the virus in the presence of an anti-adenovirus antibody increased with the number of layers, showing relatively high infectivity efficiency on cultured cells and in intraperitoneally metastatic OC [14]. Their high tropism to the liver and the induction of strong innate immune responses by macrophages and dendritic cells are another important hurdle that might limit its use.

Adeno-associated virus (AAV) can also infect a broad range of cells, like adenoviruses. AAVs have been used in treating OC delivering bevacizumab [15], Kringle 5 [16] or endostatin [17]. In a recent study, AAV9 was used in a single intraperitoneal (IP) injection to deliver albumin leader Q425R MIS (LRMIS), showing elevated and sustained serum levels of a Mullerian-inhibiting substance (MIS), which inhibited the growth of xenografts from ascites (PDXa) from patients with highly resistant recurrent OC, without overt toxicity [18].

Human papillomavirus (HPV) pseudovirions can efficiently deliver DNA into multiple cell lines, protecting it from nucleases, but pseudovirus infection of human tumours may be HPV type and tumour specific. In a study, IP injection of human papillomavirus 16 (HPV 16) pseudovirion to deliver the herpes simplex virus thymidine kinase (*HSV-TK*) gene to ovarian tumour cells was able to preferentially infect murine and human ovarian tumour cells in tumour-bearing nude mice. Subsequent administration of ganciclovir led to significant therapeutic anti-tumour effects in vitro and in vivo. This system could be used to deliver other candidate genes [19].

#### 2.1.2. Non-Viral Vectors

Viral vectors have been reported to have several problems, such as inflammatory and immune responses, insertional mutagenesis, limited loading capacity and difficult production. Non-viral vectors have a higher gene-loading capacity, lower immune response and are safer. Non-viral delivery gene systems include injection of naked DNA, transfection using liposomes, polyplexes, lipopoliplexes and nanoparticles, as well as ultrasound (US)/microbubble (MB)-mediated gene delivery [20].

Cationic lipid–DNA complexes (lipoplexes) have relatively high transfection efficiency in vitro when locally delivered at low doses. One example of this is PEI, one of the most effective non-viral gene carriers [21]. However, the application of PEI is restricted by its non-biodegradable nature and relatively high cytotoxicity. These limitations are overcome with biodegradable cationic heparin polyethileneimine (HPEI) nanogels, used to deliver several genes like survivin-T34A [22], FILIP1L [23] or gelonin toxin [24], with low cytotoxicity and high transfection efficiency and stability. Another lipoplexe is chitosan. The tumour-targeted polyethylene glycol-chitosan lactate nanoparticles with folic acid (FA) as the targeting ligand (FA-PEG-COL nanoparticles) have been effective for delivery of siRNA in OC gene therapy, thanks to its encapsulating efficiency and good protection of siRNA from serum degradation [25]. Moreover, a bioreducible disulfide-based cationic dextran system was efficient for prolonged gene delivery targeting SKOV-3 cells in vitro and in a mouse model by intravenous (iv) injection [26]. The transport of siRNA is difficult due to its hydrophilic character and its negative electric charge. The use of cationic cholesterol derivative-based liposomes is efficient as an interfering RNA (siRNA) delivery system, showing low toxicity and excellent cellular uptake and gene silencing efficiency [27].

Nanoparticles show great promise for gene delivery. *N*-[1-(2,3-dioleoyloxy)propyl]-*N*,*N*,*N*-trimethylammoniummethyl sulphate and monomethoxy poly(ethylene glycol)−poly(d,l-lactide) (DPP), a particle with very low toxicity, was enhanced with low-dosage PTX, creating a PTX-encapsulated DPP (P-DPP) nanoparticle, which increases the gene delivery and transfection efficiency of DPP nanoparticles. Furthermore PTX exerts a synergistic effect a with vesicular stomatitis virus matrix protein (*VSVMP*) gene that induces apoptosis, acting via multiple mechanisms in OC treatment. This system can efficiently inhibit the OC in vitro and in vivo [28].

Gene transfer by ultrasound-targeted microbubble destruction (UTMD) is a safe and promising technique for gene delivery, but has low gene transfection efficiency. This system produces transient pores in cell membranes and stimulates cell membrane permeabilization. In several studies, this system has been used for siRNA delivery, attempting to overcome the drawback of its low concentration at the target site. In this context, Florinas et al. combined UTMD with an arginine-grafted bioreducible polymer (ABP) and was able to synergize the advantages of each delivery systems own enhancing gene silence efficiency and siRNA transfection efficacy and VEGF protein knockdown in OC cells in vitro and in vivo [29]. There are several strategies to overcome suboptimal gene transfection efficiency such as the use of OC targeting microbubbles by conjugating LHRHa on the surface of the lipid microbubbles since it is expressed in a high percentage of OC cell lines, improving p53 gene transfection efficiency and inducing cells apoptosis by IP delivery in OC cell lines [30]; or the recombinant expression plasmid of shRNA targeting the *survivin* gene (pshRNA survivin) with a higher cell apoptosis rate (by down regulating caspase-3 and caspase-9 expression) and cell proliferation inhibitory rate [31].

#### 2.1.3. Cell-Based Vectors

**Figure 2:**
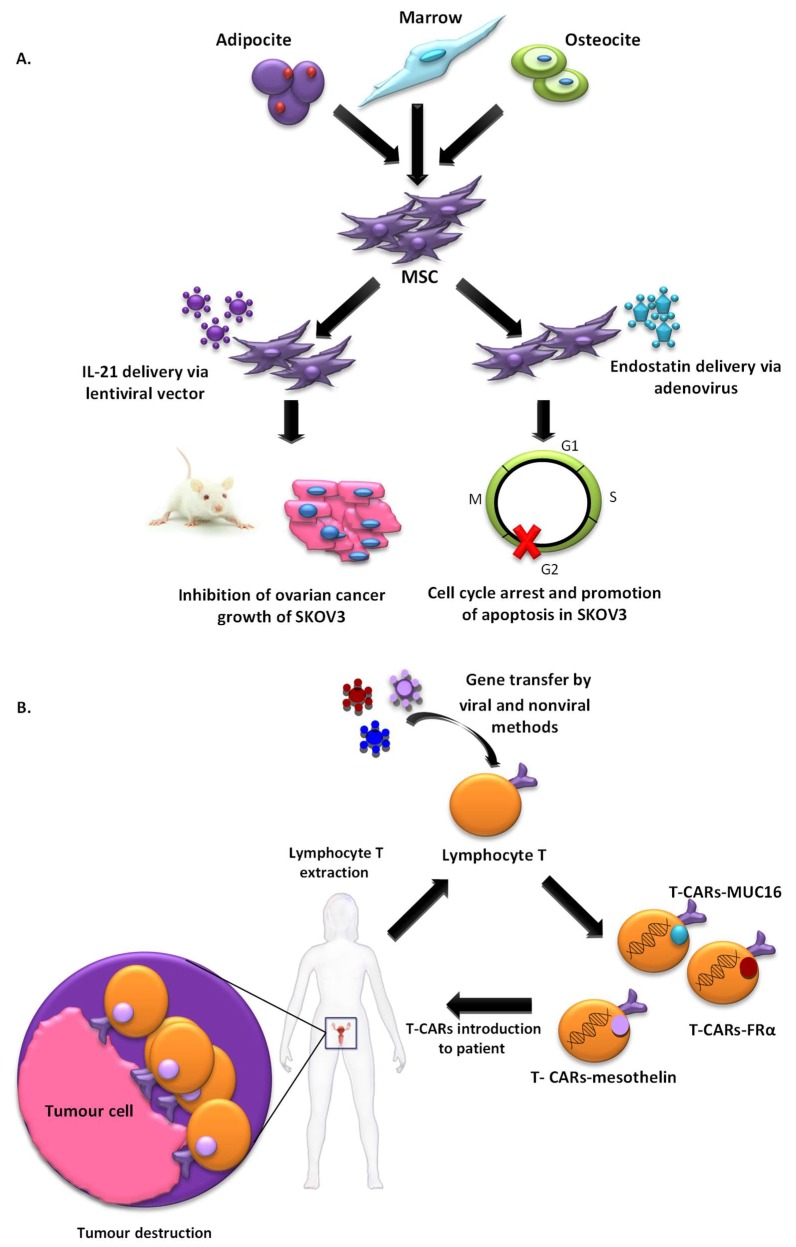
Summary of current therapies targeting ovarian cancer through the use of cell-based vectors. (**A**) MSCs as vehicles for drug delivery; (**B**) reprogramming the T cells of patients to express CARs targeting ovarian cancer.

T-cell-based immunotherapy is a therapeutic strategy that is gaining strength more and more in the treatment of cancer. A variant of this therapy that is receiving considerable attention in the investigation is chimeric antigen receptor–modified T (CAR-T)-cell therapy which was selected by the American Society of Clinical Oncology (ASCO) as the “ASCO 2018 Advance of the Year” [32]. Immunotherapy with CAR-T cells involves reprogramming the T cells of patients to express Chimeric Antigen Receptor (CAR) on their cell membrane using gene transfer technology. This receptor counts with an external target-binding domain designed to recognize a specific tumor antigen and an internal activation domain responsible for activating the T cell when the CAR-T binds its target. Second and third generation CAR-Ts have additional costimulatory domains that further enhance the immune response. In this way, the cytotoxic potential of lymphocytes T target cancer cells (Figure 2). CAR-T cells combine both T-lymphocyte activation properties and antigen specificity in a single fusion molecule. This system has been first used in patients with hematological tumours with excellent results [33], but it still needs to be improved to avoid the side effects caused. However, its use to detect solid tumours is a challenge, probably due to the characteristics of their histopathological structure and the difficulty for the infiltration of T cells in tumour sites. Despite these several challenges, its use in solid tumour, including OC, has been investigated and thoroughly reviewed by Zhu et al. [34] and Zhang et al. [35]. In this context, the most common antigens targeted by CARs in OC used in active clinical trials, include MUC16, folate receptor-α (FRα) and mesothelin, with promising preliminary results [34].

Another innovative therapeutic modality that is generating great expectations in cancer therapy is cell therapy based on the use of stem cells. The use of mesenchymal stem cells (MSCs) that have the ability to migrate to tumours as vehicles for drug delivery is an emerging strategy that could solve many of the problems generated by vectors (Figure 2). This tumour tropism is due to the repair function in which MSCs are recruited by sites of tissue injury and inflammation. Furthermore, they also have the advantage of being able to be obtained from multiple sources such as the liver, bone marrow, placenta or the umbilical cord, and could be stably amplified in vitro [36]. Zhang et al. evaluated MSCs derived from human umbilical cord for IL-21 delivery via lentiviral vector, with which they seek to obtain a more lasting expression of IL-21, to develop a therapeutic effect on SKOV3 OC xenograft-bearing nude mice. MSCs-LV-IL-21 showed an important therapeutic effect on inhibition of OC growth and safety as they do not form gross or histological teratomas up to 60 days post-transplantation in murine lung, liver, stomach and spleen [37]. In another work, MSCs derived from human bone marrow transfected with a recombinant adenovirus encoding endostatin possessed significant migratory capacity and inhibited the proliferation of SKOV3 cells by cell cycle arrest and promotion of apoptosis [38]. Dembinski et al. found that microenvironments of OC recruit MSCs after its intraperitoneally administered to participate in their stroma development, and gene-modified MSC to express IFN-β, achieved to control or eradicate ovarian tumour in tumour xenograft models, resulting in reduction of tumour growth and prolonged survival [39].

Moreover, targeting abilities of MSCs can be enhanced via the introduction of artificial receptors. In this context, Komarova et al. [40], developed, by transduction with genetically modified adenoviral vectors, a human MSC expressing an artificial receptor that binds to erbB2, a tumour cell marker (MSC-AR). MSC-AR properties were tested in human ovarian carcinoma cell line SKOV3ip1 and in vivo using transient transgenic mice that express human erbB2 in the ovarian xenograft tumour model. The binding of MSC-AR to erbB2-expressing cells was enhanced in both models suggesting that the application of this strategy enhances the efficacy of cell-based therapy [40]. However, although these preclinical studies clearly demonstrated the therapeutic benefits of using MSCs as vectors, very few clinical trials have been approved for cancer treatments. Until now, a clinical phase 1 study to determine the effects of MSCs secreting interferon beta in patients with advanced OC is being developed. The aim is to treat women with recurrent OC. For that, MSCs, isolated from healthy male donors will be genetically engineered and then be intraperitoneally administered into patients. This study, enrolled in MD Anderson with the participation of up to 21 patients, will undoubtedly bring promising results (NCT02530047) [41]. The fact that there are not many approved clinical trials can be partly due to reports that MSCs not only show a potential for malignant transformation, but may also lead to the induction of metastasis [42]. To solve this problem, a novel delivery platform based on the use of natural membrane-derived vesicles, termed nanoghosts (NGs), was developed from different biological sources. NGs developed from MSCs membrane (MSC-NGs) retain MSC surface markers and behave broadly as MSCs in relation to tumour identification capabilities in vitro and in vivo. MSC- NGs utility was proven in several types of cancers although not yet in OC [41]. MSC-NG could be a good alternative to MSCs, they are potentially safer, as they are not associated with the common risks that arise from the administration of living proliferating cells.

#### 2.1.4. Targeted Vectors for Ovarian Cancer Gene Therapy

Not only the type of vector used is important, but also the design of the vector to achieve the targeted organ. In this sense, there are different strategies, based on the use of gene regulatory elements such as promoters, or proteins that have high affinity for specific cells such as receptors that selectively eliminate cancer cells without harming healthy cells.

Based on the frequent overexpression of EGFR or its mutant in OC cells, carrying vectors guided by synthetic nano-antibodies for EGFRvIII and EGFR were developed to deliver human recombinant DNases (DNASE1, DNASE1L3, DNASE2, DFFB) into human OC cells from ascites and cultures, achieving the degradation of their genomic DNA and, consequently, cancer cell death, without affecting the healthy cells [43]. The human telomerase reverse transcriptase (hTERT) promoter can direct target gene expression to OC cells, since it is expressed in this cell type and repressed in normal tissues. In a study, hTERT was integrated in a systemic amplifier expression vector (VISA, VP16-Gal4-WPRE), enhancing transgene expression with lower toxicity than a CMV promoter. E1A, an adenoviral type 5 transcription factor that possesses various anticancer activities, was used as a therapeutic gene with this platform, being specifically targeted to OC cells and showing a significant reduction of OC cell growth in a mouse model and significantly prolonged survival compared with a control [44]. This promoter has also been used to achieve tumour-specific Thymosin β10 (*Tβ10*) gene expression, a protein that regulates actin dynamics, affecting metastasis and proliferation in many cancer cells [45]. Huang et al. demonstrated that IP administration of cationic biodegradable poly(β-amino ester) polymers may efficiently deliver diphtheria toxin subunit-A (DT-A) DNA to mice bearing ovarian tumours, using transcriptional regulation with the promoters of two genes, tumour-specific human epididymis protein 4 (*HE4*) and *MSLN*, whose activity is increased in OC cells [46]. HE4 promoter is overexpressed in a high percentage of serous and endometrioid EOC, and was used to drive the *HSV-TK* gene, showing it to be a possible treatment strategy for patients with high levels of serum HE4 [13]. Cocco et al. [47] designed a dual-targeting approach to exploit the overexpression of claudin-3/-4, the receptors for Clostridium perfringens enterotoxin (CPE), and the p16 promoter in OC cells, using an IP injection of nanoparticles (NPs) modified with the carboxy-terminal–fragment of CPE (c-CPE-NP) for the delivery of plasmid encoding for the DT-A. These particles showed themselves to be efficient in transfecting OC cells in vivo, and DT-A was effective in inhibiting ovarian tumour growth [47]. Overexpression of FRα is characteristic of OC, and could be used to direct target gene expression in human OC cells. In a recent work, FRα-targeted folate modified lipoplexes with an hTERT promoter were successfully used to drive the expression of a matrix protein (MP) of the vesicular stomatitis virus, F-LP/pMP [48]. He et al. used a FRα-targeted lipoplex (folate modified liposome, F-P-LP) with CLDN3-short hairpin RNA (shRNA) [49]. Finally, it should be noted that, given the metastasis of OC is generally confined to the abdominal cavity, OC is a good candidate for local gene therapy via IP administration [50].

### 2.2. Gene Therapy for Ovarian Cancer Treatment

Different treatment approaches have been explored in relation to gene therapy in OC: (i) tumour suppressor gene therapy that restores cell control through replacing tumour suppressor genes; (ii) oncogene inhibition strategies inactivating dominant oncogenes; (iii) suicide gene therapy by enzyme/prodrug system or activating expression of a toxin; (iv) genetic immunopotentiation by strengthening the immune response to tumour cells, augmenting the expression of tumour antigens or the production of cytokines, interleukins and growth factor; (v) antiangiogenic gene therapy (alterations in tumour vascularity to drain blood supply; (vi) multi drug resistance (MDR) associated genes strategies using genes such as PRP-4 and surviving; and (vii) oncolytic virotherapy. In Table 1, we summarize the strengths and weaknesses of these gene therapy strategies.

#### 2.2.1. Tumor Suppressor Gene Therapy

**Figure 3:**
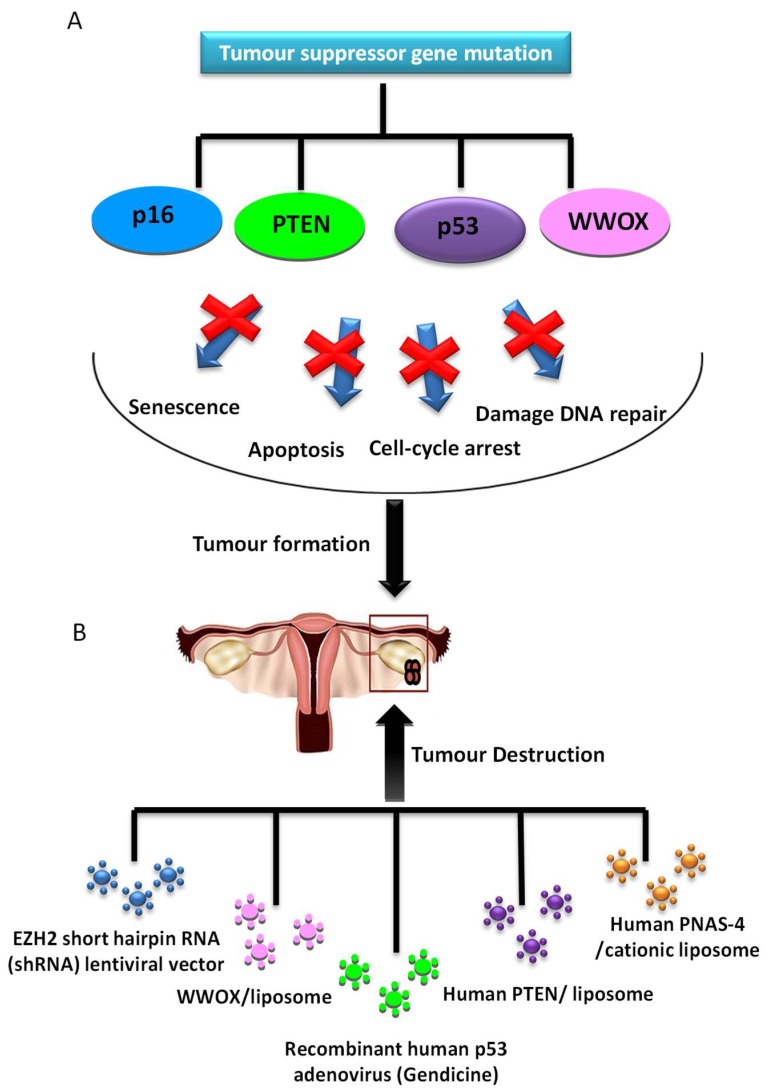
Tumour suppressor genes are involved in a wide variety of antitumour functions. (**A**) If these functions are inhibited, the tumour appears; (**B**) diverse strategies in gene therapy have tumour suppressor genes as a molecular target, enabling the recovery of gene function and tumour destruction.

Several studies of replacement of an altered tumour suppressor gene have been developed and shown antitumour efficacy (Figure 3). One of the most studied genes in cancer is p53, a protein with a wide variety of anticancer functions, thus it is involved in response to DNA-damaging, apoptosis and cell cycle and growth arrest. In a high percentage of OCs, there is a loss of p53 function. Many gene therapy approaches have focused on the role of p53 mutation, with good results both in vitro and in vivo. The transduction of wild-type p53 allows tumour proliferation inhibition [51] and increased sensitivity to cisplatin [52] and PTX [53] in preclinical research. Although, in cells with a normal *p53* gene, this therapy does not provide any additional benefit [54]. Furthermore, a recent in vitro study showed that Ad-p53 infection is an effective method to activate the apoptosis of cancer cells and re-sensitize the resistant OC cells to taxol [55], mediated by p53 upregulated modulator of apoptosis (PUMA), the direct downstream pro-apoptotic effector of p53.

However, when this strategy was studied for the first time in phase II/III trials employing an adenoviral transgene delivery system, there was no therapeutic benefit and there appeared to be complications in respect to targeting ovarian tumour cells with Ad vectors due to the lack of expression of coxsackie-adenovirus receptors, or anti-Ad antibodies in ascites [56]. However, there is new, more promising data from the use of Gendicine, a gene therapy product approved for clinical use in China in 2003 based on the injection of recombinant human Ad-p53. Its use to treat OC was studied with a response rate of 90%, and 100% resolution of the peritoneal effusion condition [57].

Moreover, overexpression of human *PNAS-4*, a pro-apoptotic gene participating in the early response to DNA damage and one of the targets of p53 tumor suppressor protein, results in a great decrease of proliferation and induces apoptosis of SKOV3 OC cells in vitro. Its iv administration through a cationic liposome showed efficient inhibition of growth and prolongation of survival in an OC mouse model, due to induction of apoptosis and inhibition of angiogenesis [58].

Furthermore, phosphatase and tensin homologue deleted on chromosome 10 (*PTEN*) is a tumour suppressor gene, frequently mutated in OC. Transfection of OC cells line with exogenous *PTEN* plasmids showed to be a good approach in increasing the expression of *PTEN* gene, which achieved significant growth suppression in OC cells by apoptosis and arrest at the G1 phase of the cell cycle. This might be due to the suppression of the PI3K/AKT pathway, besides reduction of cell migration and invasion by decreasing MMP-9 expression [59]. 

Another tumour suppressor gene is *p16*, whose upregulation in OC was demonstrated to reduce the proliferation of ovarian tumour cells by downregulation of eukaryotic translation elongation factor 1α2 protein (eEF1A2) [60]. In a recent study, Lu et al. showed that EZH2, a histone methyltransferase, negatively regulates the expression of p16, and its inhibition reduces OC cell proliferation and migration in vitro and suppresses ovarian tumour formation in vivo [61].

Finally, WW domain containing oxidoreductase (*WWOX*) gene has been identified as a tumour suppressor gene and its expression has been shown to be reduced or absent in ovarian tumours. The expression of *WWOX* gene in OC induces apoptosis and inhibits cell proliferation both in differentiated tumor cells [62] and in cancer stem cells (CSCs) [63].

#### 2.2.2. Oncofactor Inhibition Strategies

Many genes associated with the regulation of proliferation and angiogenesis are mutated in cancer, resulting in uncontrolled proliferation. These genes are a potential target for the gene silencing therapy. There are several strategies to inactivate it such as using antisense oligonucleotides binding to the target mRNA; blocking its transduction; or the use of RNA interferences (RNAi) mediated by short hairpin RNAs (shRNAs) or small interfering RNAs (siRNAs) (Table 2).

One of the most studied oncogenes is *EGFR*, related to cell migration, proliferation and differentiation, and is highly expressed in OC. In a preclinical study, siRNAs targeting *EGFR* were transferred to erythropoietin-producing hepatocellular A2 (EphA2) receptor positive OC cells by a core/shell hydrogel nanoparticle (nanogels) targeted to the EphA2 receptor, showing decreased EGFR expression levels and an increase in the sensitivity of these cells to docetaxel [64].

Moreover, the expression of Nin one binding protein (NOB1p), a protein overexpressed in OC cells and involved in protein degradation through the ubiquitin proteasome pathway (UPP), was knocked down by a lentiviral shRNA delivery system, which led to a marked reduction of the proliferation and colony formation of OC cells [65].

Metastasis associated in colon cancer 1 (*MACC1*) is upregulated in several types of cancer. Its downregulation by MACC1-specific shRNA showed inhibition of proliferation, migration capability and invasive potential of ovarian carcinoma cells. In addition, enhancement of apoptosis that was observed might be as a consequence of inhibition of HGF/Met and MEK/ERK pathways, which are widely implicated in carcinogenesis (reduced expression of Met, p-MEK1/2, p-ERK1/2, cyclin D1 and matrix metalloproteinase (MMP) protein, and an increased level of cleaved caspase 3) [66]. Metastasis-associated gene 1 (*MTA1*) also plays an important role in the invasion and metastasis in OC. Its inhibition by siRNA transfection reduced the cell invasion potential, migration and intercellular adhesion, and induced cell anoikis, a form of apoptosis in cells detached from the surrounding extracellular matrix (OC cells must acquire anoikis resistance to survive in ascites), maybe through a change of PTEN/AKT and beta 1 integrin/AKT pathway functions (since Beta 1 integrin, MMP-9 and phosphor-AKT protein levels were significantly down-regulated and PTEN upregulated) in A2780 cancer cells [67].

Cyclooxigenase-2 (COX-2) is an inducible enzyme highly expressed in OC tissues, and has an important role in the proliferation, growth, invasion and metastasis of OC cells, acting as an oncogene by stimulating proliferation and angiogenesis. Effective COX-2 silencing in human OC cells by a COX-2 specific siRNA plasmid vector [68] or a COX-2 shRNA sequence [69] inhibits cell proliferation by blocking the cell cycle in G1 phase, attenuating invasion and migration ability by a decrease in vascular growth factor, MMP-2 and MMP-9 protein expression, and suppressing the growth of OC in vitro and in vivo models.

Another gene overexpressed in ovarian epithelial carcinoma but not in normal ovarian tissue is Wilms tumor gene (*WT1*). Its expression was inhibited by WT1 antisense oligodeoxynucleotide (ASODN) that significantly inhibited cell proliferation, arrested cell cycle at G0–G1 phase and increased apoptosis in SKOV3 ovarian carcinoma cells [70].

Signal transducer and activator of transcription 3 (*STAT3*) is frequently activated in OC, being associated with the tumour formation and chemoresistance of OC. Jiang et al. used a shRNA to silence it, showing apoptosis and inhibition of cell proliferation in vitro and in vivo. Reduced tumour weight and angiogenesis were also observed. These effects could be related to STAT3 being shown to induce the expression of cleaved caspase-3 and to reduce the expression of survivin, Bcl-2, cyclin D1 and vascular endothelial growth factor [71]. In addition, the silencing of inducible factor 1-α (H1F-1α), often overexpressed in OC and associated with multiple tumour characteristics, achieved effective inhibition of cell proliferation in human OC in vitro [25].

Claudin3 (CLDN3) is a tight junction protein that is upregulated in a high percentage of ovarian tumours but not in normal ovarian tissue. Its overexpression is associated with proliferation, invasion and metastasis of ovarian tumours. Several studies have shown that downregulation of CLDN3 inhibits tumour growth in vivo and in vitro by promoting tumour cell apoptosis, inhibiting cell proliferation and reducing angiogenesis. Moreover, malignant ascites formation was inhibited in treated mice [72,73]. Huang et al. used an intratumoural injection of siRNA-lipidoid formulation [72] and Sun et al. used IP administration of shRNA-nanoparticle formulation, based on poly(lactic-coglycolic acid (PLGA) nanoparticle, a system with good biodegradability, biocompatibility and low toxicity [73].

The suppression of Notch1 activation in SKOV3 human OC cells, a protein that acts as an oncogene in OC, showed low toxicity and excellent cellular uptake and gene silencing efficiency. This study also demonstrated that this system could inhibit the growth of SKOV3 cells and promote apoptosis [27].

Downregulation of CD59, a membrane complement regulatory protein (mCRPs) that inhibits the cytolytic activity of complement, by a recombinant retrovirus encoding shRNA targeted human CD59, enhanced complement mediated cell damage, increasing apoptosis in vitro and inhibiting tumour growth in nude mice [74].

Finally, a promising tool that can be used for the inactivation of oncogenes is the Clustered Regularly Interspersed Short Palindromic Repeats (CRISPR)-caspase 9 (Cas9) genome editing technology, an editing tool that can generate deletions, insertions and replacements in the mammalian genome. He et al. targeted the OC-related DNA methyltransferase 1 gene (*gDNMT1*), an enzyme of DNA methylation whose overexpression inactivates tumour suppressor genes and which is related to tumorigenesis and resistance in OC. They found that cationic liposomal vectors (in this case a folate receptor-targeted cationic liposome, F-LP) are effective delivery systems for CRISPR-Cas9 technology, and F-LP/gDNMT1 inhibited growth of ovarian tumours in vivo, with few side effects other than a high-dose PTX injection [75].

#### 2.2.3. Suicide Gene Therapy

Suicide gene therapy is based on delivery of a gene encoding a toxin, or enzymes that convert nontoxic prodrug into toxic drugs following the administration of the inactive prodrug. The cytotoxic action is localized only in the tumour or, if applicable, in neighbouring tumour cells that have not been transduced but undergo oncolysis due to the so called “bystander effect”. This strategy is turning into tumour-targeted chemotherapy and can be categorized into two groups, direct strategies which use a gene that encodes a toxin, or indirect strategies using a prodrug [76].

The most commonly used suicide gene approach is based in herpes simplex virus-thymidine kinase (HSV-TK) followed by treatment with an antiviral drug such as ganciclovir (GCV), which is transformed into toxic metabolites by the action of thymidine kinase (TK) and other cellular enzymes, causing a failure in DNA replication and, as a consequence, cell death by apoptosis. Rawlinson et al. developed a replication-deficient adenovirus bearing the *HSV-TK* gene driven by the tumour-specific HE4 promoter. Its administration, followed by GCV treatment, increased the killing of cells by up to ten-fold in cisplatin-sensitive and resistant A2780 OC cell lines [13]. In other recent studies, the ultrasound was combined with an HSV-TK system to transfect an OC model in mice showing an enhanced tumour inhibitory effect due to apoptosis and reducing microvessel density compared with control groups [77,78]. 

Escherichia coli *cytosine deaminase* (*CD*) gene is another system with the ability to convert 5-fluorocytosine (5-FC) to 5-fluorouracil (5-FU). Sher et al. [79] developed a strategy based on the survivin promoter (protein downregulated by wild type p53 but not by mutant p53) plus a transgene amplification vector (VISA) to deliver an endostatin cytosine deaminase fusion protein (hEndoyCD) composed of an endostatin domain with antiangiogenic capacity and a CD domain that converts the 5-FC into 5-FU. This system has high tumour-specific targeting effects and induced OC cell death in vitro and in vivo without affecting normal tissues, inhibiting tumour growth and prolonging survival in mouse xenograft models. An almost synergistic cytotoxic effect in combination with cisplatin was shown [79]. Another example of suicide genes used in OC models is Escherichia coli nitroreductase (NTR), which has been shown to activate the alkylating agent CB1954 and increase survival in ovarian-infected murine models [80]. The same effect was observed using purine nucleoside phosphorylase (PNP), whose use in combination with conventional chemotherapy in multidrug-resistant OC cells showed a significant enhancement of apoptosis in OC cell lines [81].

Focusing on toxins, there are many examples that have been used in preclinical studies on OC cells. Bai et al. evaluated the antitumour activity of a recombinant plasmid DNA expressing gelonin, a toxin that causes cell death by inactivating the 60s ribosomal subunit. This plasmid was delivered by HPEI nanogels, reducing cancer cell growth and inducing apoptosis in SKOV3 cells and in mice with intraperitoneal ovarian carcinomatosis, without significant side effects [24]. In addition, DT-A is a potent inhibitor of protein synthesis. Its delivery to chemotherapy-resistant OC cells using an IP injection of nanoparticles showed an effective inhibition of OC tumour growth. This toxin was more effective than treatment with cisplatin and PTX and with minimal nonspecific cytotoxicity, and a prolonged life span compared to control mice [46,47]. Matrix protein (MP) of the vesicular stomatitis virus (VSV) inhibited the growth of tumours and enhanced the survival of mice, mediated by the induction of cancer cell apoptosis, inhibition of tumour cell proliferation and suppression of tumour angiogenesis, with a good safety profile [48]. Moreover, carbonyl reductase 1 (CBR1) reduces microvessel density and induces apoptosis. Delivering CBR1 DNA to OC cells via a polyamidoamine (PAMAM) dendrimer increased survival in mice with peritoneal carcinomatosis of OC by inhibition of dissemination and proliferation of malignant cells in mice, without significant adverse reactions [82].

#### 2.2.4. Antiangiogenic Gene Therapy

In cancer, the balance between pro- and antiangiogenic growth factors is modified in favour of angiogenesis, or new blood vessel formation from the pre-existing vasculature. A key aspect of the success of antiangiogenic treatment is maintaining optimal levels for a prolonged period with the inhibitor in circulation; gene therapy is a good strategy to achieve it.

Angiogenesis is regulated by the vascular endothelial growth factors (VEGFs) and their tyrosine kinase receptors (VEGFR-1/Ftl-1, VEGFR-2, VEGFR-3/Ftl-4), and angiopoietin growth factor and their receptors (Tie1 and Tie2). This pathway has been targeted in multiple studies, both in monotherapy and in combination therapy. Soluble Flt-1 (fms-like tyrosine kinase receptor, a potent VEGF antagonist) have shown efficacy inhibiting tumour growth and ascities formation, and increasing survival in vivo model [83]. Soluble VEGF decoy receptor (VEGF Trap) combined with PTX has enhanced the survival by complete inhibition of ascities formation and tumour metastasis, and reduced the tumour burden [84]. Several reports have studied the combined antiangiogenic gene therapy with soluble decoy VEGFRs (-1, -2 and -3, without tyrosine kinase part) delivered with adenovirus, and combination therapy has a more powerful antitumor effect than single gene therapy, suppressing tumour growth in a mouse model of ovarian carcinoma [85]. The survival is prolonged if PTX is added to combination gene therapy [86], being safe in healthy rats [87]. Furthermore, adenovirus gene therapy with soluble VEGFR2 and Ti2 reducing tumour growth and formation of ascities, enhanced reduction of ascities when gene therapy was combined with PTX and carboplatin, although in this last case, it increased the proliferation of tumour cells [88].

Other angiogenesis inhibitors that have been widely studied are angiostatin and endostatin. Adenovirus-mediated gene transfer of angiostatin or endostatin effectively inhibited malignant ascites and blood vessel formation and repressed tumour growth in OC but with transient expression [89]. To escape from the neutralization caused by humoral immune response, recombinant human endostatin adenovirus (Ad-hEndo) was encapsulated into PEG-PE cationic liposome, enhancing transfection efficiency on CAR-negative OC. Systemic administration reduced tumour growth in an established OC model, by decreasing the number of micro-vessels and increasing apoptosis of tumour cells [17]. Moreover, AAV vectors provide stable gene expression and have an excellent safety profile with persistent, long term expression. Recombinant adeno-associated virus (rAAV)-mediated delivery of a mutant endostatin (P125A-endostatin) has been shown to inhibit blood vessel formation and ovarian carcinoma growth [90]. A single intramuscular injection of rAVV-mediated delivery of K5, a potent angiogenic inhibitor, inhibited VEGF and tumour cell-induced angiogenesis in subcutaneous and intraperitoneal human OC cells in mouse models, conferring survival advantage without toxicity. Furthermore, they affect the nascent vessels more than the mature ones. This system achieved sustained levels of K5 in circulation [16]. In a more recent study, a single administration of AAVrh10.BevMab, a rhesus serotype 10 adeno-associated viral vector coding for bevacizumab, achieved persistent and high levels of bevacizumab in the peritoneal cavity with low systemic concentration, with significant reduction of OC growth through inhibition of angiogenesis, and increased survival in an OC murine model. Furthermore, combination with chemotherapeutic agents such as PTX or topotecan shows an additive effect, being more effective than monotherapy [15].

#### 2.2.5. Immunopotentiation

Strategies are based upon tumour-associated antigens and the ability of the immune system to recognize these molecules, and as such, construct encoding-known tumour antigens to elicit an immune response against it.

IL-21 has been extensively applied to significantly augment antitumour immunity in multiple murine ovarian tumour models enhancing NK cytotoxicity [91]. Hu et al. used a recombinant pIRES2-IL-21-EGFP and transfected it into CD34+ human umbilical cord blood stem cells (UCBSCs) for treatment of OC xenograft mice, with an increase in the therapeutic effect by reducing the tumour sizes and extending survival rate, markedly increasing levels of IFN-γ and TNF-α in the mouse serum, which may increase the NK cytotoxicity by upregulation of the expression of NKG2D and MIC A molecules in the tumour tissues. However, IL-21 expression was gradually decreased in the mouse tumour sites [92]. Furthermore Zhang et al. obtained a more lasting expression of IL-21 on SKOV3 OC xenograft-bearing nude mice triggering a reduction of tumour sizes, with inhibition of OC growth by downregulation expression of β-catenin and cyclin-D1, and the elevation of the aforementioned cytokines [37]. Fewell et al. developed a synthetic polymeric delivery vehicle (PPC) incorporating the anticancer cytokine IL-12 gene (*pmIL-12*) and studied its IP administration in a mouse model of disseminated OC. IP administration of pmIL-12/PPC led to elevated murine IL-12 (mIL-12) and IFN-γ levels in ascites fluid, with a significant decrease in VEGF protein that resulted in inhibition of ascites accumulation and improved survival, with no significant evidence of systemic toxicity due to IP administration (IL-12 iv administered show significant toxicities). Animal survival was improved by adding iv taxol and paraplatin chemotherapy treatment with no augmented side effects over that associated with chemotherapy alone [93].

Dendritic cells (DCs) could be loaded by self-tumour antigen and induce specific anti-tumour immunity against tumour cells carrying the target antigen. In this case, Her-2/neu was used as an antigen and was transduced into DCs by rAAV vector inducing a strong and rapid stimulation of cytotoxic T-lymphocyte (CTL) directed against OC cells [94].

#### 2.2.6. Multi-Drug Resistance (MDR)

The development of acquired drug resistance is the primary cause of chemotherapy failure in the treatment of OC, posing a major impediment to the clinical treatments. Knockdown of drug resistance-associated genes is a strategy used in OC cell lines. Inactivation of p53 by negative regulators such as murine double minute 2 (MDM2) can contribute to resistance to *p53* gene therapy. Gu et al. used a dual expression plasmid with MDM2-specific siRNA and wild-type p53, which was effective in increasing the sensitivity of cisplatin-resistant OC cells in vitro and in vivo [95].

Multidrug resistance gene 1 (*MDR1*) is overexpressed in chemoresistant OC cells. This gene encodes a membrane-bound P-glycoprotein (P-gp) that works as a drug pump, and several strategies have been developed to knock it down. Yang et al. developed hyaluronic acid-based nanoparticles that can target CD44 receptors, overexpressed on MDR OC, to deliver MDR1 siRNA, and efficiently downregulate the expression of MDR1, increasing sensitivity to PTX in MDR OC mouse models [96]. Another option is a combined therapy such as the system developed by Zhang et al., containing two anticancer drugs (doxorubicin DOX and cisplatin CIS), and two antisense oligonucleotides targeted to MDR1 an BCL2 mRNA (suppressors of cellular resistance) [97], or the use of an oncolytic adenovirus (Ad5/3) under the control of the MDR1 promoter with PTX [98].

Survivin (SVV), a member of the inhibitor of apoptosis protein (IAP) family, is associated with chemotherapy and radiotherapy resistance in OC and apoptosis inhibition of cells. This protein is upregulated in various cancers but not expressed in normal adult tissues, and silencing SVV expression induces the apoptosis of OC cells. Many therapeutic strategies targeting the *SVV* gene have been developed for OC treatment. Vivas-Mejia et al. have used siRNA to target SVV for OC and to suppress its expression [99]. Jiang et al. used adenovirus-mediated knockdown of SVV by shRNA (ad5-SVV) in cisplatin-resistant OC cells, inhibiting proliferation and invasion and inducing apoptosis, via downregulation of PCNA and MMP-2 expression and upregulation of caspase-3 expression [100]. Other studies have demonstrated a promising result in the development of vehicles such as polymeric micelles [101] and degradable HPEI nanoparticles [22] for the combined therapy of SVV siRNA and chemotherapy drugs in treatment of chemoresistant tumours.

The EGFR/extracellular signal-regulated kinase (ERK) pathway leads to tumour cell proliferation, survival and chemotherapy resistance, and it is induced by PTX, and inhibited by MicroRNA-7 (miR-7) by downregulating EGFR expression. Cui et al. have developed a dual-drug-delivery system based on biodegradable polymer nanoparticles that do not have these problems, to simultaneously deliver PTX and miR-7. Despite the results of products of this combination, this therapy has some limitations, because it was not able to completely eradicate the tumours [102].

Another way to act on chemoresistance is through the clock gene, an important regulator of the inherent circadian rhythm in mammals. Formations of a heterodimer transcription factor complex with another protein activate the expression of many genes regulating metabolism, eating, physiology and behaviours. Sun et al. found that suppressing the Clock gene expression can induce autophagy and can upregulate the cisplatin-induced apoptosis in OC cells in vitro. Furthermore, enhancing cisplatin chemosensitivity by the inhibition of the expression of resistant genes like *MRP2* or *P-gp*, by combined therapy cisplatin and interfering Clock expression, have superior cytotoxicity to cisplatin alone [103]. In addition, glucose regulated protein 78 (GRP78) is involved in cell survival during endoplasmic reticulum stress, and contributes to development of chemoresistance. Its silencing by siRNA transfection increases the sensitivity to PTX in OC cell line (HO-8910) by induction of cancer cell apoptosis [104]. Moreover, the TNF-related apoptosis-inducing ligand (TRAIL) is a protein with the ability to induce apoptosis in a broad range of cancer cells, without affecting normal cells, but has instability in vivo and resistance to cancer cells. A study showed that the use of retrovirus encoding *TRAIL* gene inhibited growth of drug-resistant A2780/DDP ovarian carcinoma cells in vitro via a caspase-activated apoptotic mechanism, and in combination with cisplatin-enhanced anticancer activity in vitro and in a xenograft nude mouse model. This may be an efficient approach to treat drug-resistant OC [105].

#### 2.2.7. Oncolytic Virotherapy

Cancer virotherapy is a strategy in which viruses are modified to preferentially replicate in tumour cells and lead to cell death, through targeted alterations in the cancer cells, such as *p53* mutation, viral deletion, tissue-specific transcriptional control, or tumour-specific receptors. Furthermore, oncolytic viruses (OVs) can be genetically designed to deliver therapeutic genes as suicide genes.

The viral glycoproteins hemagglutinin (H) of measles virus (MV), responsible for receptor attachment and rigger in cell-entry, can be genetically engineered to use any cell surface receptor of choice for cell entry. Designed Ankyrin repeat proteins (DARPin) domains allow the generation of oncolytic viruses with double specificity, simultaneously targeting HER2 and EpCAM54 (a cancer stem cell marker), handling intratumoural variation of antigen expression and targeting simultaneously CSCs and the tumour mass, showing oncolytic potential in a disseminated OC xenograft model in mice, furthermore, showing the superior efficacy of bispecific over monospecific viruses [106].

Reoviruses kill ovarian-cancer cells in vitro, this effect is reduced by ascites due to the presence of neutralizing antibodies (NAb). However, cytotoxicity can be restored using a combination of lymphokine-activated killer and dendritic cells (LAKDC) as carriers, which protect the virus from NAb in the ascites [107].

The combination of oncolytic herpes simplex viruses (HSV) with immunostimulatory cytokines has recently been studied in attempts to increase its efficacy. Genetically engineered HSV were transformed to express the cytokines IL-12, having a cytotoxic effect in an OC cell line, and its IP administration in mice models had a longer survival and lower rates of peritoneal metastasis, with an increased CD8+ T-cell immune response [108]. HF10 intraperitoneal injection, a highly attenuated variant of the HSV type 1 (HSV-1), decreased tumour size in a murine OC model, and its combination with a Granulocyte–macrophage colony-stimulating factor (GM-CSF) can elicit immune response, with higher antitumoural effects [109].

The VSV is one of the most potent oncolytic viruses, but presents neurotoxicity and induction of Nab; limitations are overcome by pseudotyping VSV with the glycoprotein of the lymphocytic choriomeningitis virus (LCMV)—resulting in virus VSV-GP. Selectivity replication in cancer cells by this virus is determined by reduced antiviral defense due to aberrations in the type I interferon (IFN) system, which is very common in tumour cells. However, in OC cell lines, most cells have the IFN response intact, and VSV-GP oncolysis can be enhanced by combination with an inhibitor of the interferon response, such as Ruxolitinib, inhibitor of Jak1 and Jak2. Dold et al. showed that IP treatment with VSP-GP was able to infect, replicate in and kill most OC cell lines tested, but tumour remission in mice was only temporary. However, the combined therapy with the Jak1/2 inhibitor ruxolitinib enhanced efficacies compared with monotherapy, and there was no increase in virus toxicity [110].

The combination of Myxoma virus (MYXV) and cisplatin produced a significant improvement in overall survival in a mouse model of disseminated OC, and reduced secretion of cytokines immunosuppressives by CD14+ myeloid cells [111]. The recombinant oncolytic adenovirus ZD55-MnSOD (an antioxidant enzyme with tumour suppressor activity) enhances cisplatin-mediated growth suppression and apoptosis in OC cells, in vitro and in vivo, so the combination therapy of cisplatin and ZD55-MnSOD results in an improved survival rate, compared to monotherapy [112].

Different studies describe the use of suicide gene therapy in combination with virotherapy for ovarian carcinoma treatment. Hartkopf et al. developed a combined strategy based on a recombinant MeV armed with a bifunctional suicide fusion gene that encodes for CD and uracil phosphoribosyltransferase (MeV-SCD), that enhance the sensitivity of chemoresistant cancer to 5-FU by its conversion into the toxic metabolite 5-fluorouridine monophosphate (5-FUMP). This combined therapy showed an effective infection and lysis of human OC cell lines and primary tumour cells derived from malignant ascites of OC patients, and 5-FC significantly enhanced the antineoplasic activity of MeV-SCD [113].

## 3. Clinical Trials

According to The Journal of Gene Medicine Clinical Trial site (http://www.abedia.com/wiley/indications.php), there are a total of 1688 clinical trials for cancer gene therapy, representing 65% of the total clinical trials based on gene therapy compiled in this registry. Clinical trials have explored the feasibility and effectiveness of several gene therapy treatment strategies for OC listed before, including tumour suppressors, suicide genes and oncolytic virotherapy (Table 3). To date, the clinical trial registry contains 2176 trials about OC (Source: clinicaltrials.gov), and 31 clinical trials involving gene therapy have been registered.

Immunopotentiation is the strategy with the most clinical trials in gene therapy of OC made so far. As we can see in Table 2, delivery of cytokines to the tumour (NCT00473954, NCT00137865, NCT02480374), administration of tumour vaccines based on tumour-associated antigens (TAAs) (NCT00381173, NCT03073525) or T-Cells genetically engineered to express T-cell receptors reactive against mutated neoantigens (NCT01567891, NCT02869217, NCT02366546, NCT02457650, NCT01583686) were used. IL-12 is one of the most widely studied cytokines in this sense, due to its potent immune-modulatory properties and its ability to inhibit tumour angiogenesis. The latest studies have been directed at the evaluation of EGEN-001, a system composed of a human IL-12 plasmid and a delivery system polyethyleneglycol-polyethyleneimine-cholesterol (PCC) that facilities its delivery in vivo. Research groups have brought about two phase I [115,116] and one phase II trials [117] in patients with recurrent OC. In the phase I trials, EGEN-001 was administered IP alone or in combination with chemotherapy, showing to be safe and feasible with low grade and manageable side effects and disease response in some patients. The phase II trial evaluated the toxicity and antitumour activity of EGEN-001 administered IP at the 24 mg/m^2^ dose, in patients with platinum-resistant recurrent ovarian, fallopian tube or primary peritoneal cancer. Specific toxicity was similar to that found in phase I studies. More frequent adverse events presented in the 20 treated patients were grade 1/2, including nausea, vomiting, pain, fatigue and anemia, but EGEN-001 was less tolerated than in the previous studies. Of the 16 patients evaluable for response, seven had stable disease and nine had progressive disease, with no partial or complete response, showing that EGEN-001 in monotherapy had limited activity in platinum-resistant EOC patients. Another more recent study evaluated the administration of EGEN-001 at a higher dose (36 mg/m^2^) in combination with liposomal doxorubicin, which presents the ability to modulate the immune system in various ways, was initiated [118]. This phase I trial showed promising results, with a clinical benefit (partial responses and stable disease) of 57.1% in the 14 patients with measurable disease, achieving 28.6% of partial responses and 57.1% of stable disease at dose level 3.

Reintroduction of tumour suppressor genes has been widely studied in preclinical studies, and *p53* is one of the most extensively studied. In the clinical trial registry we can find that several studies about the insertion of the *p53* gene are being carried out (NCT00003450, NCT00003588, NCT00003880, NCT02435186), alone or in combination with chemotherapy.

Numerous groups have evaluated suicide-based gene therapy in OC (NCT00964756, NCT00005025, NCT02576665). The suicide gene most commonly investigated has been HSV/TK with several clinical trials completed. Kim et al. studied a new strategy to enhance the infectivity of a RGD-modified adenovirus (Ad5-SSTR/TK-R-GD) expressing *HSV-TK* gene and a somatostatin receptor (SSTR) used as a non-invasive strategy to assess gene transfer through nuclear imaging [119]. This phase I study was conducted in a cohort of 12 patients, showing the safety of using this vector intraperitoneally, with limited clinical toxicities (manageable constitutional or pain symptoms), related to the dose. In relation to effectiveness, no partial or complete responses were observed, although five patients had stable disease 1 month after treatment and Ca-125 levels decreased in three. Furthermore, a patient had a delayed and durable complete response, being clinically free of disease 25 months after treatment.

Over the last two decades, several clinical trials based on oncolytic virotherapy have been developed. One of the main approaches has been the use of serotype-5 conditionally-replicative adenoviruses (CRAds), which has shown problems in relation to the non-expression of its receptor, the CAR, on the surface of OC cells. This has led to the development of novel strategies to enhance the infectivity of adenoviruses cells [120,121,122]. In a phase I study in 10 recurrent OC patients, Kim et al. used Ad5/3-Δ24, a CRAd which incorporates a modification that alters the tropism towards serotype-3 adenoviral receptors, and a deletion for selective replication in Rb-p16-deficient tumour cells [120]. Its IP administration daily for three consecutive days at dosages up to 1 × 10^12^ vp, showed it to be safe, with mild and manageable vector-related toxicities. Clinical efficacy analyses showed that 75% of patients had RECIST-defined stable disease and 37.5% had a decrease in CA-125 levels, but a marked anti-adenoviral antibody response was also noted. These results are similar to those of other trials evaluating other strategies of increased infectivity of CRAds in OC, such as the study carried out by Koski et al. in 21 patients with a variety of solid tumours including four patients with OC [121]. A serotype 5/3 chimeric oncolytic adenovirus expressing GMCSF was administrated and one of the OC patients had RECIST-defined stable disease and a slight decrease in CA-125 levels.

## 4. Future Directions

Despite all the efforts made to treat and cure OC, approximately 80% of patients diagnosed with ovarian epithelial cancer will relapse after standard first-line treatment, which includes platinum-based and taxane-based chemotherapy [123]. These suggest that CSCs may play an important role in OC and cannot be ignored. It is believed that CSCs are responsible for tumour initiation and contribute to chemo and radioresistance, which explains relapse and resistance in OC. Ovarian cancer stem cells (OCSCs) are a subpopulation of OC cells with self-renewing ability, and can differentiate into heterogeneous tumour cell types. Some markers have been found in OCSCs, including CD44, CD47, CD133, ALDH1, CD24, CD117 (c-kit), epithelial cell adhesion molecule (EpCAM), and SOX2, with a heterogenic expression [124]. Moreover, ALDH plays an important role in the resistance of OC to chemotherapeutics, especially in OC stem cells [125]. In a recent study, Shaomin and Guang correlated ALDH2 mutation to the low incidence and mortality of OC in East Asian women [126]. Furthermore, an overexpression of several OCSCs markers have been found in residual ovarian tumours after treatment with chemotherapy [124]. Thus, OCSCs markers have been considered as useful therapeutic targets to minimize drug resistance and the tumour relapse of OC. Targeting some of these markers has been studied in several works [127]. In addition, certain substances have been shown to be effective in treating OCSCs, such as metformin, niclosamide (commonly used against parasites) and salinomycin (antibiotic isolated from *Streptococcus albus* bacteria and has been tested in humans too); hence, these drugs could be used in combination with gene therapy to improve the response to treatment. Some studies support the ability of OCSCs to adhere and to spread on mesothelial layers, showing characteristics of mesenchymal cells [127].

Another approach is the use of anti-angiogenic drugs such as Bevacizumab or Trebananib, since there is a link between CSCs and angiogenesis [128]. Many inmunotherapeutic strategies can be used to target OCSCs, and some of them have already been researched in some works: NK cell, cancer therapeutic vaccine (a CD177/CD44 vaccine), monoclonal antibody immunotherapy (catumaxomab, which can bind to CD133 and EpCAM) and the blockade of immune checkpoints.

In the field of gene therapy applied on OCSCs, Chimeric antigen receptor (CAR)-T cells, genetically modified T cells, could be used to target these specific antigens on OCSCs, and have been used to target CD133 in OC cell lines [129]. Moreover, Long et al. studied its elimination by truncated Bid (tBid), a potent inducer of cell apoptosis, delivery by the Cre/LoxP system, targeting CD133, a cell surface marker of OCSCs. They constructed two recombinant adenoviruses, the first facilitates the expression of the Cre in CD133+ CSCs, which cut-off LoxP sequences from the second, thus allowing the overexpression of tBid, driven by the CMV promoter, in CD133+ CSCs. This system induced the apoptosis and inhibited growth of OCSCs in vitro and in vivo, in addition to increasing the cytotoxic effect of cisplatin [130]. Furthermore, it has been shown that OCSCs expressed several genes related to primordial germ cells, germinal lineage, and pluripotency, such as Nanog, Oct4 and Sox2; therefore, their involvement in the manifestation of OC is not excluded [131]. The gene therapy with shRNA or siRNA targeting Nanog has already shown some promising therapeutic potential [132].

Furthermore, the low transduction efficiency of the vector within the tissue, and the creation of neutralizing antibodies to viral vectors by the immune system is a great limitation of the actual gene therapy systems. New non-viral vectors for OC are increasingly investigated. The use of nanoghosts, vesicles derived from the natural membrane as a vehicle for gene delivery, represents an innovative and promising approach for targeted OC. Although there are several types of vesicles, such as exosomes and red blood cells, that are being manipulated for the delivery of genes and/or drugs, MCSs are undoubtedly the best option for ovarian cancer since they have inherent targeting capabilities and can be produced using a technologically scalable and pharmaceutically applicable process [42].

## 5. Conclusions

No effective therapies exist for patients with advanced-stage OC. This malignancy is confined to the abdominal cavity, offering the option of a localized treatment with protection of the organs out of the peritoneal cavity and gene therapy could be a promising strategy. This review showed the development of multiple systems for OC treatment based on gene therapy with encouraging preclinical results. However, the translation to humans has not yet shown a significant clinical benefit due to, among others, the lack of efficient vectors. This fact has led to the development of numerous new vectors, many of them from non-viral origin. To improve tumour targeting, specific tumour promoters, tumour gene targets and markers have been identified. They were used to achieve effective penetration within advanced tumour masses and to minimize toxicity in normal tissues. These improved vectors are beginning to be analysed in phase I trials, and constitute one of the main fields of research for future works. On the other hand, the use of gene therapy as monotherapy has not been as successful as had been expected. Combining gene therapy with chemotherapy and radiotherapy has been shown to improve the effectiveness and safety of the treatment by reducing the dosages. This could also be applicable to the combination with other emerging therapeutic strategies, such as targeting molecular pathways with angiogenesis inhibitors (bevacizumab) or Poly ADP Ribose Polymerase (PARP) inhibitors (olaparib), and immunotherapy (ipilimumab, an anti-CTLA-4, or nivolumab, an anti-PD-1 agent). Finally, OCSCs have been shown to contribute to cancer progression, metastasis, chemoresistance and recurrence, and their discovery has opened a new area of research in OC. Several OCSCs markers have been suggested with a high variability of their phenotypical features in the different studies. Even so, there is no doubt that these markers will open the way to the targeting of OCSCs to minimize the drug resistance and tumour relapse.

In conclusion, there is still much work to be done in order to reach the full potential that gene therapy can offer for the treatment of OC. More phase I and II clinical trials are needed to investigate current preclinical strategies so as to translate this powerful strategy to the clinic.

## Figures and Tables

**Table 1:** Strengths and weaknesses of various gene therapy strategies for ovarian cancer treatment.

Therapeutic Strategy	Gene/System	Strong Points	Weak Points
Tumor Suppressor gene	*p53*	Altered gene in a high percentage of OC. The use of Gendicine (a recombinant human Ad-p53) has been approved in the treatment of OC in China. Several clinical trials are currently ongoing.	Not useful in cells with a normal *p53* gene. No therapeutic benefit in the first clinical trials due to the use of a wrong delivery system.
	*WWOX*	Promising results in vivo Inhibition of proliferation and promotion of apoptosis in OCSC.	It has not been evaluated in clinical trials in OC.
Oncofactor inhibition strategies	*EGFR*	Gene widely studied in cancer. Its use increases the sensitivity to chemotherapy	It has not been evaluated in vivo or in clinical trials in OC.
	CLDN3	Several strategies of silencing have been studied in OC (siRNA and shRNA). Promising results in vivo, with inhibition of malignant ascites formation.	It has not been evaluated in clinical trials in OC.
Suicide gene therapy	HVS-TK	Strategy widely studied in gene therapy for cancer. Several clinical trials are currently ongoing. Promising results in vivo and in clinical trials. Bystander effect.	Not phase 2 or 3 clinical trials published.
	DT-A	Promising results in vivo, with minimal cytotoxicity.	It has not been evaluated in clinical trials in OC.
Antiangiogenic gene therapy	VEGFRs	Promising results in vivo, with inhibition of ascities formation. Synergist effect in combination with chemotherapy.	It has not been evaluated in clinical trials in OC. The work showed increased proliferation of tumour cells with the use of VEGFR2 and Ti2 in combination with PTX and carboplatin.
	Endostatin	Promising results in vivo. Clinical trials have been carried out in other types of cancer.	Transient expression due to humoral immune response if adenovirus is used as delivery system. It has not been evaluated in clinical trials in OC.
Genetic immunopotentiation	IL-12	Potent immune-modulatory properties and ability to inhibit tumour angiogenesis. Promising results in vivo. Several clinical trials are currently ongoing or have been completed. It has showed to be safe and feasible in phase I trials.	Poor clinical benefits in the completed phase II trial.
	CAR-T cell	Possibility of target any specific tumour antigen. Promising preliminary results in clinical trials in OC.	Important side effects caused in patients.
Multi-Drug Resistance	*MDR1*	Several strategies have been developed to knockdown it. Its silencing enhances sensitivity to anticancer drugs. Promising results in vivo.	It has not been evaluated in clinical trials in OC.
	*Survivin*	Several strategies targeting this gene have been developed, including combined therapy with anticancer drugs. Promising results in vivo.	It has not been evaluated in clinical trials in OC.
Oncolytic virotherapy	VSV	Can be genetically designed to deliver therapeutic genes. A phase I trial is currently ongoing.	Neurotoxicity and Induction of neutralizing antibodies. IFN response intact in most OC cells, blocking virus replication.

**Table 2:** Most frequent oncofactor inhibition approaches used in ovarian cancer.

Gene	Function in Ovarian Cancer	Silencing Strategy	Model	Ref.
*EGFR*	Cell migration, proliferation and differentiation	siRNA carried in nanogels	In vitro	[64]
*NOB1*	Protein degradation through ubiquitin proteasome pathway (maturation of the 20S proteasome)	shRNA carried in a lentiviral system	In vitro	[65]
*MACC1*	Regulation of MET, which is involved in cellular growth and migration, angiogenesis, invasion and metastasis	shRNA plasmid	In vitro	[66]
*MTA1*	Component of histone deacetylase 1 involved in transcriptional regulation. May enhance cell invasion, migration, adhesion and anoikis-resistance.	siRNA plasmid	In vitro	[67]
*COX2*	Prostaglandin synthesis, involved in stimulation of proliferation and angiogenesis in cancer	siRNA and shRNA plasmids	In vitro and in vivo	[68,69]
*WT1*	Proliferation and differentiation of the urogenital system	ASODN carried in liposomes	In vitro	[70]
*STAT3*	Regulation of multiple oncogenes and suppressor gene expressions involved in cell proliferation and apoptosis and angiogenesis	shRNA carried in DOTAP-cholesterol liposomes	In vitro and in vivo	[71]
*H1F-1α*	Transcriptional regulator of the adaptive response to hypoxia by activation of genes involved in cell proliferation and migration, angiogenesis, apoptosis and glucose metabolism	siRNA through FA-PEG-COL nanoparticles	In vitro	[25]
*CLDN3*	Component of tight junction (TJ) of epithelial cells and cancer cells, so is involved in invasion and metastasis	siRNA carried in lipidoid molecules, shRNA carried in PLGA-NPs, shRNA carried in F-P-LP	In vitro and in vivo	[72,73]
*NOTCH1*	Cell development, proliferation, differentiation and apoptosis.	siRNA carried in cationic cholesterol derivative-based liposomes	In vitro	[27]
*CD59*	Inhibition of cytolytic activity of complement	shRNA carried by a recombinant retrovirus	In vitro and in vivo	[74]
*gDNMT1*	DNA methylation, involved in tumorigenesis, relapse and resistance of ovarian cancer.	CRISPR-Cas9 delivered by F-LP	In vitro and in vivo	[75]

**Table 3:** Current ovarian cancer gene therapy clinical trials available in [114] “clinicaltrials.gov” until May 2018 using the terms “ovarian cancer” and “gene therapy” as key words.

Therapeutic Strategy	Intervention	Clinical Trial Reference	Phase	Year (First–Last Posted)
**Suicide gene therapy**	HSV-TK + GCV Vector: Adenovirus (Ad5.SSTR/TK.RGD)	NCT00964756	Phase 1	2009–2013
	HSV-TK + GCV Vector: Vector producer cells (VPC)	NCT00005025	Phase 2	2003–2013
	CD + 5-FC Vector: Toca 511, a purified retroviral replicating vector encoding a modified yeast *CD* gene	NCT02576665	Phase 1	2015–2018
**Tumour suppressor gene**	Inserting the *p53* gene Vector: Adenovirus (Ad5CMV-p53)	NCT00003450	Phase 1	2003–2009
	Inserting the *p53* gene Vector: Adenovirus (Ad5CMV-p53)	NCT00003588	Phase 1	2004–2013
	Inserting the *p53* gene + chemotherapy (PTX and carboplatin) Vector: Adenovirus (SCH-58500)	NCT00003880	Phase 2 Phase 3	2004–2015
	Inserting the *p53* gene + chemotherapy (cisplatin and PTX)	NCT02435186	Phase 2	2015–2015
**Oncolytic virotherapy**	LOAd703 (an oncolytic adenovirus serotype 5/35 encoding immunostimulatory transgenes: TMZ-CD40L and 41BBL) + chemotherapy or gemcitabine	NCT03225989	Phase 1 Phase 2	2017–2018
	Recombinant carcinoembryonic antigen (CEA)-expressing measles virus (MV-CEA) and oncolytic measles virus encoding thyroidal sodium iodide symporter (MV-NIS)	NCT00408590	Phase 1	2006–2018
	Vesicular Stomatitis Virus expressing Human Interferon Beta and Sodium-Iodide Symporter (VSV-hIFNbeta-NIS)	NCT03120624	Phase 1	2017–2018
**Immunopotentiation**	EGEN-001 (IL-12 Plasmid Formulated With PEG-PEI-Cholesterol Lipopolymer) + chemotherapy	NCT00473954	Phase 1	2007–2013
	EGEN-001 (IL-12 Plasmid Formulated With PEG-PEI-Cholesterol Lipopolymer)	NCT00137865	Phase 1	2005–2013
	GEN-1 (IL-12 Plasmid Formulated With PEG-PEI-Cholesterol Lipopolymer) + chemotherapy (PTX and carboplatin)	NCT02480374	Phase 1	2015–2018
	NYESO-1(C259) transduced autologous T cells	NCT01567891	Phase 1 Phase 2	2012–2018
	TBI-1301 (Autologous T cells engineered to express a T cell receptor (TCR) targeting NY-ESO-1) + cyclophosphamide	NCT02869217	Phase 1	2016–2017
	TBI-1301 (Autologous T cells engineered to express a T cell receptor (TCR) targeting NY-ESO-1) + cyclophosphamide ± fludarabine	NCT02366546	Phase 1	2015–2017
	Autologous T cells engineered to express a T cell receptor (TCR) targeting NY-ESO-1 + cyclophosphamide + fludarabine	NCT02457650	Phase 1	2015–2016
	TBI-1201 (MAGE-A4-specific *TCR* gene transduced T lymphocytes) + cyclophosphamide ± fludarabine	NCT02096614	Phase 1	2014–2017
	Gen modified lymphocytes with *MOv-gamma chimeric receptor* gene (*MOv-PBL*) + IL-2	NCT00019136	Phase 1	2003–2015
	TCR-Transduced PBL (T-Cells Genetically Engineered to Express T-Cell Receptors Reactive Against Mutated Neoantigens)	NCT03412877	Phase 2	2018–2018
	Anti-mesothelin CAR transduced PBL (retroviral vector that contains a chimeric T cell receptor (CAR) that recognizes mesothelin) + cyclophosphamide, fludarabine and aldesleukin	NCT01583686	Phase 1 Phase 2	2012–2018
	Anti-hCD70 CAR PBL (Transducing PBL with a chimeric antigen receptor (CAR) that engages CD70) + cyclophosphamide, fludarabine and aldesleukin	NCT02830724	Phase 1 Phase 2	2016–2018
	ZYC300 (vaccine which encodes the cytochrome P450 family member, CYP1B1, a known human tumor-associated antigen) + cyclophosphamide Vector: PGL-encapsulated plasmid DNA	NCT00381173	Phase 1	2006–2013
	Vigil (vaccine composed of autologous tumor cells which are transfected extracorporeally with a plasmid encoding for the gene for *GM-CSF*, an immune-stimulatory cytokine, and a bifunctional short hairpin RNA that targets furin, convertase responsible for activation of both TGβ1 and β2) + Atezolizumab	NCT03073525	Phase 2	2017–2018
	ALVAC(2)-NY-ESO-1 (M)/TRICOM vaccine + IDO1 inhibitor	NCT01982487	Phase 1 Phase 2	2013–2013
	ALVAC(2)-NY-ESO-1 (M)/TRICOM vaccine + sirolimus + GM-CSF	NCT01536054	Phase 1	2012–2018
	ALVAC(2)-NY-ESO-1 (M)/TRICOM vaccine + sargramostim	NCT00803569	Phase 1	2008–2011
	atezolizumab ± guadecitabine ± CDX-1401 vaccine (a vaccine composed of a human mAb specific for DEC-205 fused to the full-length tumor antigen NY-ESO-1)	NCT03206047	Phase 1 Phase 2	2017–2018
	p53MVA vaccine (modified vaccinia virus ankara vaccine expressing tumor protein p53) + gemcitabine hydrochloride	NCT02275039	Phase 1	2014–2018
	p53MVA vaccine + Pembrolizumab	NCT03113487	Phase 2	2017–2018
	p53 peptide vaccine + ISA-51 + IL-2 ± GM-CSF	NCT00001827	Phase 2	1999–2017
